# PTRF suppresses the progression of colorectal cancers

**DOI:** 10.18632/oncotarget.9424

**Published:** 2016-05-18

**Authors:** Fengyun Wang, Yongqiu Zheng, Matthew Orange, Chunlin Yang, Bin Yang, Jiong Liu, Tao Tan, Xiangxue Ma, Tin Chen, Xiaolan Yin, Xudong Tang, Hua Zhu

**Affiliations:** ^1^ Gastroenterology Department, Xiyuan Hospital, China Academy of Chinese Medical Sciences, Beijing, China; ^2^ Institute of Basic Medical Sciences of Xiyuan Hospital, China Academy of Chinese Medical Sciences, Beijing, China; ^3^ Department of Physical Education and Human Performance, Central Connecticut State University, New Britain, CT, USA; ^4^ Department of Surgery, Davis Heart and Lung Research Institute, The Ohio State University Wexner Medical Center, Columbus, OH, USA

**Keywords:** PTRF, mTOR, colorectal cancer, progression

## Abstract

As a key component of caveolae structure on the plasma membrane, accumulated evidence has suggested that Polymerase I and Transcript Release Factor (PTRF) plays a pivotal role in suppressing the progression of human malignances. However, the function of PTRF in the development of colorectal cancers is still unclear. Here we report that the expression of PTRF is significantly reduced in tumor tissues derived from human patients with colorectal cancers, and that the downregulation of PTRF correlates to the advanced stage of the disease. In addition, we found that the expression of PTRF negatively regulates the tumorigenic activities of colorectal cell lines (Colo320, HT29 and CaCo2). Furthermore, ectopic PTRF expression caused significant suppression of cellular proliferation, and anchorage-independent colony growth of Colo320 cells, which have the lowest expression level of PTRF in the three studied cell lines. Meanwhile, shRNA mediated knockdown of PTRF in CaCo2 cells significantly promoted cellular proliferation and anchorage-independent colony growth. In addition, *in vivo* assays further revealed that tumor growth was significantly inhibited in xenografts with ectopic PTRF expression as compared to untreated Colo320 cells, but was markedly enhanced in PTRF knockdown CaCo2 cells. Biochemical studies revealed that overexpression of PTRF led to the suppression of the AKT/mTOR pathway, as evidenced by reduced phosphorylation of AKT, mTOR, and downstream MMP-9. Thus, these findings, for the first time, demonstrated that PTRF inhibits the tumorigenesis of colorectal cancers and that it might serve as a potential therapeutic target for human colon cancer patients.

## INTRODUCTION

In the United States, colorectal cancer ranks as the 3^rd^ most frequent cause of cancer death amongst women and men, and has a probability of diagnosis at some point during life of 4.7% and 5%, respectively [1]. The American Cancer Society projected that 1,665,540 new cancer cases would occur in the U.S. during 2014, that 136,830 of these would be colorectal cancer, and that this specific disease would cause 50,310 deaths [2]. Globally, 693,900 colorectal cancer deaths occurred in 2012 [3]. Despite detailed knowledge of the occurrence and mortality rates of colorectal cancer, an understanding of its underlying molecular mechanisms remains unclear.

The function of PTRF in cancer is disputed. There have been multiple studies that support its role as a tumor suppressor, showing that its expression decreases with certain types of lung and breast cancers [4–6], and that lack of expression is associated with tumor growth and metastasis in prostate cancer [7–9]. Directly contrasting these observations, separate studies have found PTRF to promote progression and resistance to treatment in breast cancer, pancreatic cancer, glioblastomas, and colorectal cancer [10–13]. Such contrasting observations have led multiple groups to postulate that the role of PTRF in cancer varies with the specific type and with the specific stage [14]. Thus, it is vital that the detailed role of PTRF in specific cancer types be investigated.

Although a potential role for PTRF as an oncogene or a tumor suppressor has been investigated in different types of tumors, it has not been previously tested whether PTRF plays a role in the development of colorectal cancer. Here we show that PTRF expression is significantly reduced in tumor tissues. Retrospective study analysis further showed that PTRF negatively correlates with the TNM stage and with lymph node metastasis of colorectal patients. In *in vitro* studies, ectopic expression of PTRF in Colo320 cells, which have a relatively lower level of PTRF expression, significantly suppressed cellular proliferation. Additionally, PTRF inhibited *in vivo* tumor growth in a nude mice model. Meanwhile, shRNA knockdown confirmed the tumor suppressive role of PTRF in CaCo2 cells, which express the protein at a higher level. Molecular analysis further revealed that PTRF inhibited the activation of the AKT/mTOR pathway, indicating that PTRF may control the progression of colorectal cancers by downregulating this signaling pathway. Our study suggests that PTRF inhibits the tumorigenesis of colorectal cancer cells and that it might serve as a potential therapeutic target for human patients with colorectal cancers.

## RESULTS

### PTRF expression is decreased in tumor tissues derived from patients with colorectal cancers

**Figure 1:**
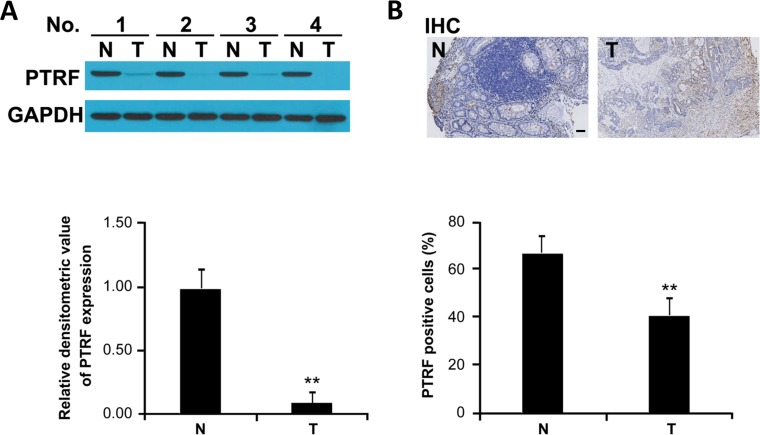
PTRF expression is decreased in human colorectal cancer tissues **A**. PTRF expression in colorectal cancer tissues (T) and adjacent non-cancerous tissues (N), derived from human patients, was detected by Western blot analysis; GAPDH was used as the internal control. Western blotting results were quantified using ImageJ software and summarized in *lower panel*. ** *p* < 0.01. **B**. Immunohistochemistry of representative samples showed that expression of PTRF is reduced in colorectal cancer. Scale bar: 100 μm.

To determine the potential role of PTRF in progression of human colorectal cancer, we first examined the expression of PTRF in cancer tissues from colorectal cancer patients as compared to adjacent, non-cancerous tissues from those same patients. Western blotting analysis showed that protein expression of PTRF was significantly decreased in cancer tissues (Figure 1A).

### Downregulation of PTRF in tumor tissue correlates with disease stages of colorectal cancer patients

In order to further establish the correlation of PTRF expression with clinical prognosis, pathological slides from colorectal cancer patients were stained with PTRF antibody. Consistent with immunoblotting results, we found that PTRF is highly expressed in non-cancerous cells, while expression in paired tumor cells is greatly reduced (Figure 1B). As summarized in Table 1, expression of PTRF in colorectal cancer tissues was negatively correlated with the TNM stage (*p = 0.0179*) and histologic stage (*p = 0.0443*), but was not related to other clinicopathological characteristics, such as age (*p = 0.901*), gender (*p = 0.817*), or disease localization (*p = 0.923*).

**Table 1:** Clinicopathologic features of colorectal cancer patients

variables	Number	PTRF expression		*P* value
		High	low	
**Age**				
**<60**	13	4	9	0. 901
**≥60**	25	7	18	
**Gender**				
**Male**	22	6	16	0. 817
**Female**	16	6	10	
**Tumor location**				
**Colon**	16	5	11	0. 923
**Rectum**	22	7	15	
**Tumor size**				
**<4**	17	5	12	0.879
**≥4**	21	7	14	
**Stage**				
**I**	4	3	1	0.0443
**II**	9	3	6	
**III/IV**	25	6	19	
**TNM Classification**				
**T**				
**T1/T2**	6	4	2	0.0179
**T3/T4**	32	8	24	
**N**				
**N0**	14	7	7	0.0315
**N1/N2/N3**	24	5	19	

### PTRF regulates proliferation, migration and invasion of colorectal cancer cells

**Figure 2:**
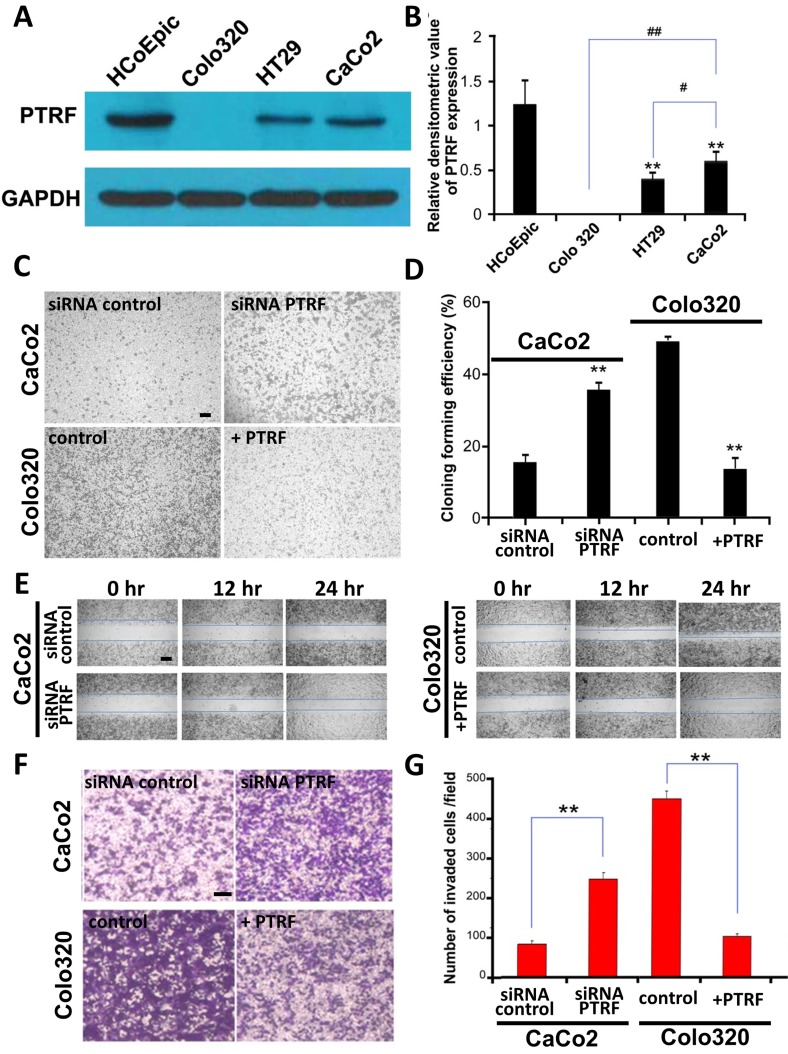
Expression of PTRF is differentially reduced in colorectal cancer cells and may be involve in regulating tumor progression **A**. PTRF was reduced in three colorectal cancer cell lines, Colo320, HT29, CaCo2, as compared to normal control cells, HCoEpiC. The anchorage independent colony formation **C**., **D**., cell migration **E**. and invasion **F**., **G**. abilities of Colo320 cells were the highest amongst the three colorectal cancer cell lines. Six random fields were photographed **C**., **F**. and summarized **D**., **G**. in each group. Data presented as Mean ± SD, n ≥ 3 independent experiments. * p < 0.05, ** p < 0.01. Scale bar: 100 μm.

In order to investigate a potential role of PTRF in regulation of tumor progression, several colorectal cell lines(Colo320, HT29, CaCo2) and normal colorectal epithelial cells (HCoEpiC) were studied. Consistent with our patient observations, levels of PTRF were reduced in colorectal cancer cell lines as compared to HCoEpiC cells (Figure 2A and 2B). Among cancer cell lines, PTRF expression was lowest in Colo320 cells and highest in CaCo2 cells (Figure 2A). We then tested whether the differential expression of PTRF would lead to differences in proliferation, migration, and invasion among these cell lines. Anchorage independent colony formation analysis showed that Colo320 cells established more colonies than CaCo2 cells (Figure 2C and 2D, p *< 0.01*). A similar trend was observed upon evaluation of cell migration (Figure 2E) and invasion (Figure 2F and 2G, p *< 0.01*). As our observations were consistent with a previous study from another group [15], we hypothesized that PTRF is involved with the regulation of proliferation, migration, and invasion of colorectal cancer cells.

In order to further test our hypothesis, we stably overexpressed PTRF (+PTRF) in Colo320 cells, as they have the lowest endogenous expression of the protein amongst the three cell lines, and treated CaCo2 cells, which have the highest expression, with shRNA knockdown (siRNA PTRF). After generation of cell lines, we then performed proliferation, anchorage-independent colony growth, migration, and invasion experiments by comparing our genetically modified cells to parental cells. As shown in Figure 2C and 2D, Colo320 cells overexpressing PTRF displayed a reduced cell proliferation rate as compared to that of parental cells and empty vector transfected cells, while siRNA PTRF cells proliferated significantly faster than control CaCo2 cells. Furthermore, RFP-PTRF cells showed reduced migratory (Figure 2E) and invasive (Figure 2F and 2G) capacities, while siRNA PTRF cells were more aggressive than the control CaCo2 cells in terms of migration (Figure 2E) and invasion (Figure 2F and 2G). These results suggested that PTRF does, indeed, regulate cell proliferation, migration, and invasion in colorectal cancer cells.

### PTRF suppresses the activation of AKT/mTOR pathway in colorectal cancer cells

**Figure 3:**
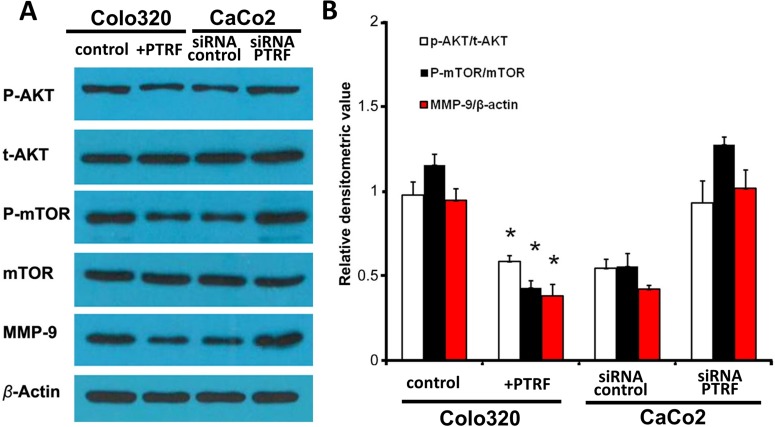
PTRF inhibits the AKT/mTOR signaling pathway in colorectal cancer cells **A**. Overexpression of PTRF led to inhibition of the AKT/mTOR signaling pathway in Colo320 cells as evidenced by downregulation of phosphorylation of its key components, such as AKT, mTOR, and downstream MMP-9. Knockdown of PTRF led to activation of the same pathway in CaCo2 cells. **B**. Density of western blotting bands were quantified and summarized. Data presented as Mean ± SD, n ≥ 3 independent experiments. * p < 0.05.

In order to determine the molecular mechanisms underlying PTRF mediated tumor suppression, the AKT/mTOR signaling pathway was tested by western blotting in our established cells lines. Previous studies have reported mTOR to promote cell survival [16, 17], and have indicated a role for PTRF in regulation of the AKT/mTOR signaling pathway in other diseases [18]. As shown in Figure 3A, ectopic expression of PTRF resulted in the deactivation of the AKT/mTOR pathway, as it inhibited the phosphorylation of AKT, mTOR, and downstream MMP-9 (Figure 3A). In CaCo2 cells, knockdown of PTRF expression significantly enhanced the activation of the AKT/mTOR pathway (Figure 3A and 3B). Thus, we demonstrated that PTRF negatively regulated the activation of the AKT/mTOR pathway, which may account for its tumor suppressive function.

### PTRF inhibits the growth of colorectal cancer cells *in vivo*

**Figure 4:**
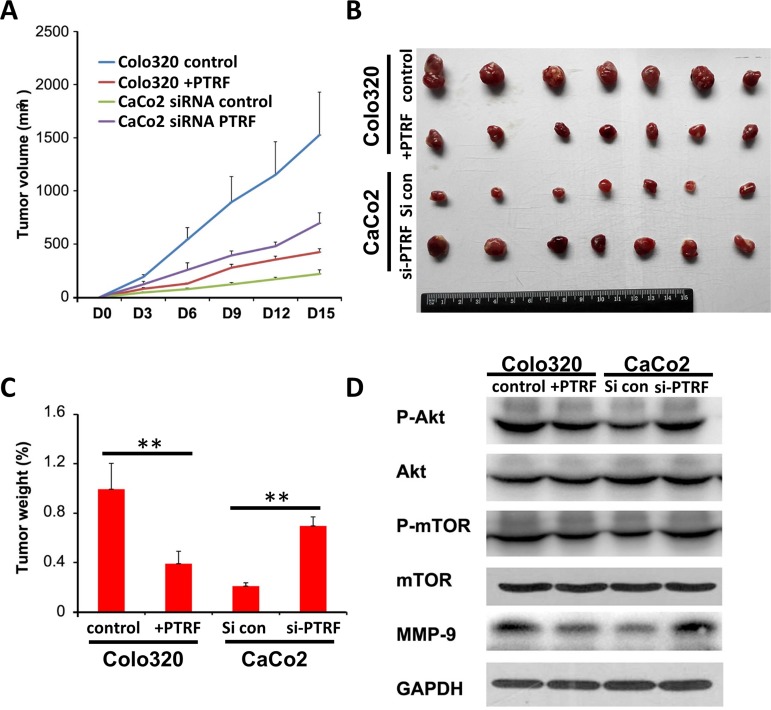
PTRF inhibits ***in vivo*** tumor growth of colorectal cells. Six-to-eight week old female BALBc/nude mice were subcutaneously injected in the flank with 1×10^6^ Colo320 or CaCo2 cells in a volume of 100 μl. The mice were sacrificed and the tumors were dissected and weighted **B**., **C**. on the 15^th^ day after inoculation. Tumor size was measured by using a digital caliper every three days after injection **A**.. **D**. AKT/mTOR signaling pathway proteins derived from xenografts were tested by Western blotting. Data presented as Mean ± SD, five mice for each group. ** p < 0.01.

To further test the role of PTRF in the regulation of colorectal cancer cell growth *in vivo*, nude mice were inoculated with control Colo320 cells (control) or +PTRF Colo320 cells, and control CaCo2 (si con) or si-PTRF treated CaCo2 cells. The volume of tumor xenografts were then monitored every 3 days and, at day 15 post-injection, they were removed for photographing and weight measurement. The results showed that overexpression of PTRF significantly suppressed growth of xenografts in nude mice, as measured 6 days after inoculation (Figure 4A), and reduced the final weight of tumors (Figure 4B and 4C). Similarly, in CaCo2 cells, knockdown of PTRF significantly promoted tumor growth in nude mice as compared to control CaCo2 cells (Figure 4A-4C). After sacrifice, xenografts were dissected out the animals and subjected to Western blot analysis of the AKT/mTOR pathway. As shown in Figure 4D, overexpression of PTRF inhibited AKT/mTOR signaling, while knockdown of PTRF activated AKT/mTOR signaling in xenografts. Therefore, these results suggested that PTRF can regulate colorectal tumor growth *in vivo*.

## DISCUSSION

The role of PTRF as an oncogene or a tumor suppressor may be specific to the cell and/or tissue type, thus accounting for the controversy surrounding the topic. In this study, we reported that PTRF expression is significantly decreased in tumor tissues of colorectal cancer patients. Detailed analysis further revealed that PTRF is significantly correlated with TNM stage and disease stage. Given that the PI3K/AKT/mTOR pathway is important to metabolic, growth, and proliferative processes of the cell, genetic variation and abnormal expression or activity of its components has drawn the attention of those studying cancer biology, colorectal cancer included. Expression of experimentally mutated mTOR genes in cultured cells promotes tumorigenicity [19], and mutations to the genetic sequences of pathway components that modulate mTOR activity, such as PI3K, PTEN, and AKT have been detected in colorectal cancers [20–26]. Rapamycin and everolimus, inhibitors of mTORC1, display promising antitumor effects, decreased proliferation, increased apoptosis, and reduced tumor size in experimental models [27–29]. However, inhibitors that simultaneously target mTOR and PI3K, have shown more promising and clinically relevant results [30, 31].

As to the role of PTRF in colorectal cancer progression, we found that ectopic expression of PTRF inhibited the activation of the AKT/mTOR pathway, suppressed proliferation, migration, and invasion, and reduced *in vivo* tumor growth of colorectal cancer cells with a relatively low PTRF expression. The inhibitory role of PTRF was also identified *via* PTRF knockdown. However, whether PTRF directly depends on repressing the AKT/mTOR pathway in order to inhibit colorectal cancer progression remains to be determined. In addition, since there are many factors that are involved in AKT/mTOR signaling pathways, it will be also important to identify what specific factors are involved in PTRF mediated tumor suppression.

Development of multidrug resistance amongst cancer cells consistently complicates treatment options and patient outcomes. Resistance appears to be achieved by the export of drugs from the cell by P-glycoprotein (P-gp), concentrated within invaginated lipid rafts, caveolae [32–35]. PTRF is essential to the formation of caveolae [36], and, by extension, the drug resistance conferred by P-gp. Increased expression of P-gp and PTRF, as well as an increased number of lipid rafts, is found in drug resistant breast cancer [13] and glioblastoma cells [12]. PTRF knockdown in resistant breast cancer cells decreases the number of lipid rafts and increases sensitivity to adriamycin treatment [13]. Knockdown in resistant glioblastoma cells significantly reduces expression of both caveolin-1 and P-gp, while increasing the effectiveness of various cancer drugs [12].

In our study, we have observed that PTRF inhibits the proliferation, migration, and invasion of colorectal cancer cells. Future studies need to define the role of PTRF in the regulation of chemo-resistance in colorectal cancer cells and in colorectal cancer stem cells. Taken together, our findings implicate PTRF as an essential inhibitor during colorectal cancer development and identify it as a potential biomarker for human patients with colorectal cancers.

## MATERIALS AND METHODS

### Patients and tissue samples

All research involving human participants was approved by the Ethics Committees of Xiyuan Hospital China Academy of Chinese Medical Sciences. Written informed consents have been obtained from all participants. Thirty-eight pathologically diagnosed biopsy specimens and paired adjacent normal tissues were acquired from patients with colorectal cancers. The patients' clinicopathological characteristics are detailed in Table 1. Biopsy samples of these 38 patients were cut into 5 μm slices for PTRF immunohistochemical staining and then used for the retrospective cohort analysis.

### Cell cultures

Normal colorectal epithelial cell line HCoEpiC was obtained from ScienCell, and cultured in Colonic Epithelial Cell Medium that recommended by ScienCell at 37°C in a humidified atmosphere with 5% CO2. Human colorectal cancer cell lines (Colo320, HT29 and CaCo2) were cultured in RPMI-1640 medium containing 10% fetal bovine serum (Gibco) at 37°C in a 5% CO2 air atmosphere as mentioned before.

### Immunohistochemical staining

Biopsy samples were fixed in 4% formalin, and embedded in paraffin. Tissue slices that were 5-μm thick were cut for H&E staining and examined under a microscope. Immunohistochemistry was performed using the Vectastain ABC Kit (Rabbit IgG, Vector Laboratories). The PTRF antibody was used as primary antibody. Slices were developed with DAB and counterstained with hematoxylin. For immunohistochemistry assessment, the entire tissue section was scanned and scored by two independent pathologist. The scores were calculated as Lai *et al* described [37] and higher than 1 was considered positive.

### Western blotting

Protein of tissue samples or cell lines was extracted by RIPA buffer (150 mM NaCl, 50 mM Tris-Cl, 1 mM EGTA, 1% Triton X-100, 0.1% SDS and 1% sodium deoxy cholate, pH 8.0). The protein concentration was determined using protein assay solution (Bio-Rad). Equivalent proteins were denatured in protein loading buffer, loaded onto 10% SDS-PAGE gels, and subsequently transferred to polyvinylidene difluoride (PVDF) membranes (Millipore, Billerica, MA) by electroblotting. The PVDF membranes were blocked with 5% nonfat milk in TBST buffer for 1 h and incubated overnight at 4 ^°^C with antibody against PTRF (Abcam, 1:2000), Akt (Cell Signaling, 1:2000), pAkt (Ser473) (Cell Signaling, 1:1000), mTOR (Cell Signaling, 1:2000), pmTOR (Ser2448) (Cell Signaling, 1:1000), MMP-9 (Cell Signaling, 1:2000), GAPDH (Cell Signaling, 1:2000) and β-actin (Sigma, 1:5000). Signals were detected using ECL detection reagent (Pierce, Inc) following the manufacturer's instructions.

### Establishment of stably transfected cells

The cDNA for PTRF was amplified by PCR. RFP-PTRF was constructed by inserting PTRF cDNA into mRFP-C1 (invitrogen) using EcoRI and KpnI. For knockdown studies, the target sequence in the PTRF cDNA was selected, the appropriate shRNA oligo was cloned into pU6-mRFP vector to generate pU6-mRFP-shPTRF. This plasmid has a red fluorescent protein expression cassette under control of a separate promoter that acts as a marker of cell transfection. All plasmids were confirmed by direct nucleic acid sequencing. Cells were transfected with specific plasmid or empty vector using Lipofectamine 2000 according to the manufacturer's instructions (Invitrogen, USA) and retained in medium containing 10% FBS.

### Anchorage independent colony formation

To examine anchorage-independent cell growth, we used a colony formation assay in soft agar. First, 3 ml of DMEM/10% FCS containing 0.5% low-melting agarose was placed in six-well dishes as a bottom support layer. After the bottom layer was solidified, 0.5 ml of top agar-medium mixture (DMEM/F12, 10% FCS, 0.33% agar) containing 10^3^ cells was added, and the dishes were incubated at 37°C for two weeks. The numbers of colonies were counted under a microscope.

### Cell migration assay

Cells were grown to 90% confluence in a 6-well plate. Cells were subjected to a scratch wound assay with 200-μL pipette tips. Time dependent cell migration was recorded using a Zeiss Axiovert 200 phase-contrast microscope at 0 h, 12 h and 24 h post-injury.

### Cell invasion assay

Transwell inserts that precoated with Matrigel (8 μm pore; Corning Costar) were placed into the 24-well plate, a total of 1×10^5^ cells in 0.5% FBS-medium were seeded in the upper chamber and 10% FBS-medium were added in the lower chamber. After 24 h incubation, cells on the top surface of the insert were removed by wiping with a cotton swab. The cells that had invaded the bottom side of the membrane were fixed with 4% formaldehyde and stained with DAPI. The numbers of invasive cells were obtained by counting five fields per membrane and represented the average of three independent experiments.

### Xenograft mouse model

Six-to-eight week's old female BALBc/nude mice were purchased from Laboratory Animal Center of Xiyuan Hospital and maintained in the cages under sterile conditions with a specific pathogen-free environment. All experimental protocols described in this study were approved by the Ethics Review Committee for Animal Experiment of Xiyuan Hospital. Cells were subcutaneously injected in the flank of mice (1×10^6^) in a volume of 100 μl at the time of inoculation [38]. Tumor size was measured by external caliper measurement every three days after injection, and tumor volume was calculated as 1/2 × (tumor length) × (tumor width)^2^ [39]. The mice were sacrificed and the tumors were dissected and weighted on the 15^th^ day after inoculation.

### Statistical analysis

All statistical analyses were performed using Graphpad Prism V.5.00 software (GraphPad Software, San Diego CA, USA). Statistical significance was determined at *p < 0.05*, and tests were two sided. Pearson's χ^2^ test or Fisher's exact test were used to test the difference between PTRF expression and clinicopathologic characteristics. Comparison between two groups for statistical significance were performed with unpaired Student's t test. For more groups, one-way ANOVA followed by Neuman-Keuls post hoc test was used.
